# The scaffold protein Ajuba suppresses CdGAP activity in epithelia to maintain stable cell-cell contacts

**DOI:** 10.1038/s41598-017-09024-4

**Published:** 2017-08-23

**Authors:** J. J. McCormack, S. Bruche, A. B. D. Ouadda, H. Ishii, H. Lu, A. Garcia-Cattaneo, C. Chávez-Olórtegui, N. Lamarche-Vane, V. M. M. Braga

**Affiliations:** 1 Molecular Medicine, National Heart and Lung Institute, Imperial College London, SW7 2AZ London, UK; 2 Cancer Division, Faculty of Medicine, Imperial College London, SW7 2AZ London, UK; 3 Cancer Research Program, Research Institute-McGill University Hospital Centre and Department of Anatomy and Cell Biology, McGill University, H4A 3J1 Montreal, Quebec Canada; 4 Department of Biochemistry and Immunology, Institute of Cell Biology, Federal University of Minas Gerais, Belo Horizonte, Brazil

## Abstract

Levels of active Rac1 at epithelial junctions are partially modulated via interaction with Ajuba, an actin binding and scaffolding protein. Here we demonstrate that Ajuba interacts with the Cdc42 GTPase activating protein CdGAP, a GAP for Rac1 and Cdc42, at cell-cell contacts. CdGAP recruitment to junctions does not require Ajuba; rather Ajuba seems to control CdGAP residence at sites of cell-cell adhesion. CdGAP expression potently perturbs junctions and Ajuba binding inhibits CdGAP activity. Ajuba interacts with Rac1 and CdGAP via distinct domains and can potentially bring them in close proximity at junctions to facilitate activity regulation. Functionally, CdGAP-Ajuba interaction maintains junctional integrity in homeostasis and diseases: (i) gain-of-function CdGAP mutants found in Adams-Oliver Syndrome patients strongly destabilize cell-cell contacts and (ii) CdGAP mRNA levels are inversely correlated with E-cadherin protein expression in different cancers. We present conceptual insights on how Ajuba can integrate CdGAP binding and inactivation with the spatio-temporal regulation of Rac1 activity at junctions. Ajuba provides a novel mechanism due to its ability to bind to CdGAP and Rac1 via distinct domains and influence the activation status of both proteins. This functional interplay may contribute towards conserving the epithelial tissue architecture at steady-state and in different pathologies.

## Introduction

Integrity of epithelial tissues relies on the ability to maintain robust cell-cell junctions. These must be able to withstand a host of challenges from the outside environment, whilst maintaining a level of plasticity to remodel contacts where necessary in response to specific cues^1, 2^. Understanding the intricate regulation of cell-cell adhesive complexes can offer insights into developmental and homeostatic processes. Moreover, it may uncover potentially clinically relevant targets. Much evidence exists implicating the improper regulation of E-cadherin adhesive receptors and junctional components in tumourigenesis as well as other disorders^2, 3^.

Amongst the most important players governing epithelial cell-cell contacts and downstream signalling are the Rho GTPases. These are molecular switches that, when activated, can interact with a range of effector proteins to bring about specific downstream responses^4^. Rac1 activation is vital for the formation and maintenance of E-cadherin contacts, including actin recruitment and remodelling at sites of contact. The precise spatiotemporal activation of Rac1 by cadherin engagement is of paramount importance for junction homeostasis^5^. Yet, how this is achieved is not well understood. Regulators such as the Rho Guanine nucleotide exchange factors (GEFs) and GTPase activating proteins (GAPs) facilitate the activation and inactivation of specific GTPases, respectively, in a temporal and spatially restricted manner. However, the identification of Rac1- specific GAPs that operate at epithelial contacts has been less well-defined^5^.

Here, we have identified the Cdc42 GTPase-activating protein CdGAP (also known as ARHGAP31) as a novel regulator of cell-cell contact maintenance. CdGAP regulates both Rac1 and Cdc42 activities, but not RhoA^6, 7^. There is compelling evidence to support an essential role for CdGAP in various diseases. Truncating mutations in the terminal exon of the *CdGAP* gene are found in patients with the developmental disorder Adams-Oliver syndrome (AOS), which leads to prematurely truncated proteins with enhanced GAP activity and results in migration defects^8, 9^. The syndrome is characterised by congenital absence of skin (to various extent on the skull) and transverse limb defects, from lack of distal phalanges, entire digits or whole limbs^10^ and cardiac and pulmonary complications^11^. Furthermore, CdGAP single nucleotide polymorphisms (SNPs) are associated with coronary artery diseases^12, 13^ while embryonic vascular development is severely compromised in CdGAP knockout mice^14^. Recent studies support the notion that CdGAP is a positive modulator of breast cancer metastasis via two potential mechanisms: (i) CdGAP expression acts as a co-repressor of E-cadherin transcription^15^ and (ii) CdGAP levels are increased in ErbB2-transformed mammary tumour explants where it participates in TGF-β-stimulated epithelial-to-mesenchymal transition, cell migration and invasion^16^.

At the cellular level, CdGAP modulates cell migration and spreading, lamellipodia formation, focal adhesion turnover and matrix rigidity-sensing^6, 17–20^. CdGAP has not been formally implicated in the regulation of epithelial cell-cell contacts. In addition to the transcriptional regulation of E-cadherin^15^, we have previously shown that CdGAP inactivates Rac1 at cell-cell contacts^21^ but the functional implications are unknown. Here we identify CdGAP as a negative regulator of mature junctions in epithelial cells, via a functional interplay with the LIM domain-containing protein Ajuba^22^. Ajuba is an actin binding and bundling protein^23^ that localises to focal adhesions and cell-cell contacts^24, 25^. Despite possessing no catalytic activity itself, Ajuba regulates Rac1 activity to stabilize cadherin adhesion^23^ or promote wound healing^25^, respectively. In keratinocytes, Ajuba interacts with both active and inactive Rac1 and modulates active Rac1 levels at sites of cell-cell contacts^23^.

Here we show that CdGAP must be inactivated in order to preserve mature junctions. A direct interaction with Ajuba maintains a pool of CdGAP localized at cadherin adhesion sites and attenuates significantly the disruption of junctions caused by CdGAP expression. Overall, these findings present an elegant mechanism whereby Rac1 activity can be precisely controlled at cell-cell contacts via an actin binding protein that retains and inactivates a Rac1 GAP at junctions. Thus, the biochemical and functional interplay between CdGAP and Ajuba represent a novel pathway to modulate Rac1 function at epithelial contacts in homeostasis and potentially different pathologies.

## Results

**Figure 1:**
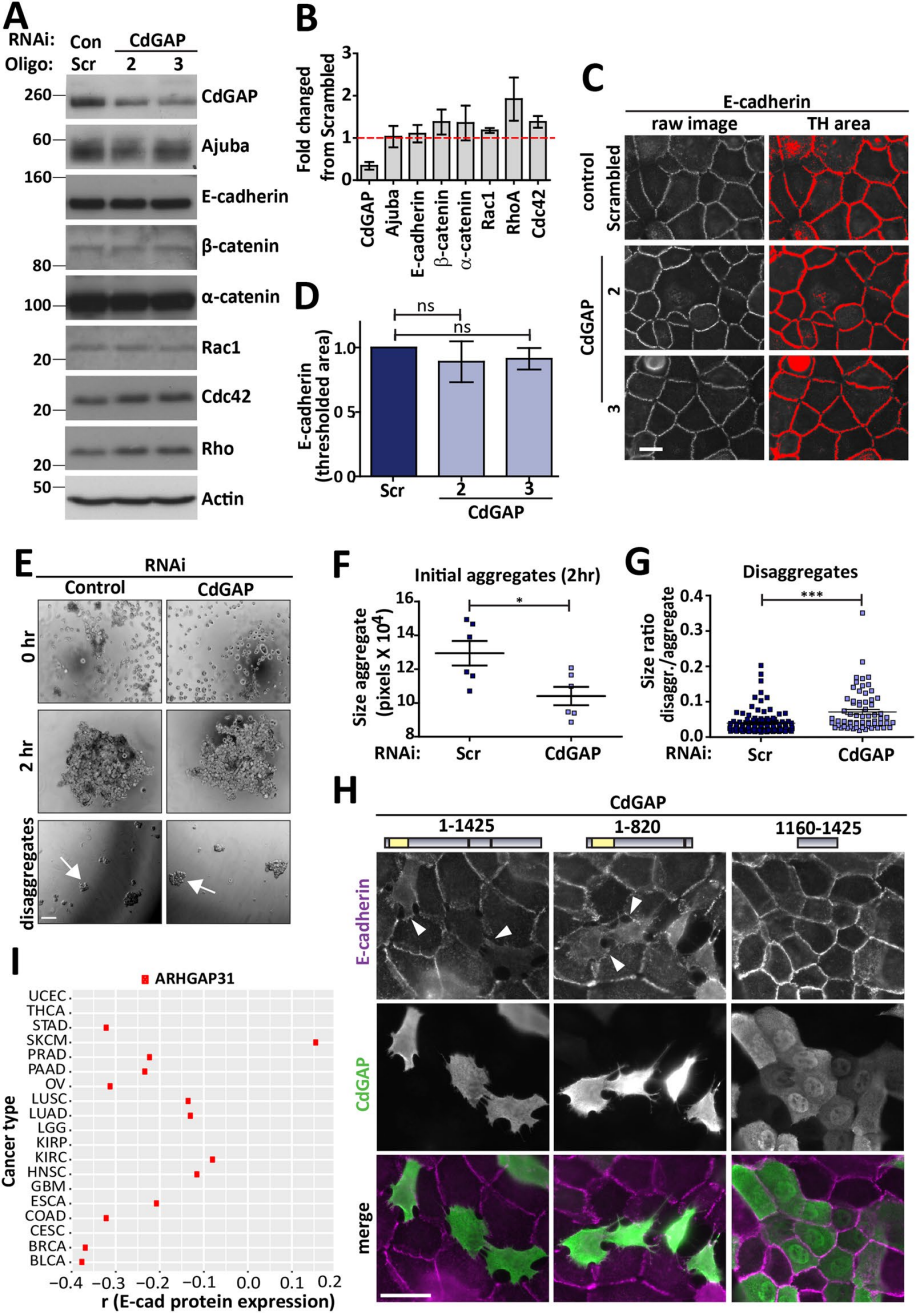
CdGAP regulates adherens junction maintenance. (**A**–**D**) Keratinocytes grown in low calcium medium were depleted of CdGAP using two specific oligonucleotides (oligo 2 and oligo 3) for 48 hours. (**A**) Cell lysates were prepared and probed for proteins shown on the right hand side of the panels. Molecular weight markers are shown on the left. (**B**) Graph shows the total levels of junctional proteins in cells depleted of CdGAP relative to scrambled or non-targeting siRNA-treated control cells. (**C**,**D**) Confluent monolayers were induced to form junctions for 30 minutes, fixed, and stained for E-cadherin (raw image). Images were collected from a wide-field microscope and processed for E-cadherin levels at the junction (TH area). (**D**) Graph shows the mean thresholded area E-cadherin for each condition. Values are expressed relative to the control non-targeting oligonucleotide. (**E**–**G**) Keratinocytes were dissociated into a single cell suspension and placed into at least six hanging drops per condition in a humid chamber (0hr). Cells were allowed to aggregate for 2 hours (2 hr), dissociated by pipetting and all the resulting disaggregates imaged (disaggregates). The size of initial aggregates (**F**) and all disaggregates formed post-disruption were measured. The size of disaggregates was normalised to initial aggregate size (**G**) (representative experiment shown; see Figure S1). (**H**) Keratinocytes maintained in standard calcium medium were transfected with myc-CdGAP, myc-CdGAP_1–820_ or myc-CdGAP_1160–1425_ constructs and expressed for 8 hours as indicated. Cells were fixed and stained for E-cadherin (purple) and myc-tagged proteins (green); merged images are shown in the bottom row. Arrowheads show junctions with interrupted E-cadherin staining. (**I**) Correlation between E-cadherin protein levels and ARHGAP31 (red squares) mRNA expression levels in different tumour types. The X-axis indicates the spearman correlation coefficient and Y-axis indicates individual cancer type (see Table S1 for cancer types abbreviations); only correlations with adjusted p value < 0.05 are shown. Scale bar = 25 µm (**C**), 100 µm (**E**) or 50 µm (**H**). Statistical significance was assessed using one-way ANOVA with Bonferroni’s multiple comparison test (**D**), T-test (**F**) or Mann-Whitney test (**G**) and standard error of the means is shown. *P ≤ 0.05, ***P ≤ 0.001, Ns = not significant. N = 3 (**A**–**D**, **H**) or N = 2 (**F**–**G**).

To determine whether CdGAP is involved in the formation of cell-cell contacts in human keratinocytes, CdGAP was depleted using two oligonucleotides (Fig. 1A–D). CdGAP-depleted and control cells had no significant changes in the levels of Rho GTPases, Ajuba and other junctional proteins (Fig. 1A,B). Newly formed junctions were induced for 30 minutes and upon quantification (Fig. 1C,D), CdGAP depleted cells showed slightly reduced levels of E-cadherin at contacts compared to controls, but this did not reach significance. Furthermore, when allowed to aggregate in hanging droplets, depleted cells formed aggregates smaller than controls (Fig. 1E,F), suggesting that CdGAP appears to play a role in junction formation in keratinocytes in suspension. In spite of similar E-cadherin levels at junctions, mechanical disruption showed that CdGAP depleted keratinocytes had more stable cell-cell contacts (Fig. 1G, Figure S1). These results are in line with previous reports that cadherin junctional levels are not a bona-fide predictor of the strength or stability of cell-cell adhesion^26^. Thus, the smaller sized initial aggregates formed in suspension are more resistant to disruption by mechanical stress.

Furthermore, mature junctions found in cells maintained in standard calcium medium were highly sensitive to CdGAP up-regulation. Expression of CdGAP full-length or a truncated CdGAP containing amino acids 1–820 (CdGAP_1–820_) induced large gaps at junctions between expressing as well as non-expressing neighbouring cells (Fig. 1H). Expression of the CdGAP C-terminus alone, CdGAP_1160–1425_, which harbours no recognised structural domain, had no effect on E-cadherin junctions. This construct also localized in the cytoplasm and more frequently in the nucleus when compared to wild-type or CdGAP_1–820_. However, the nuclear localization was not sufficient to disrupt contacts, excluding a sequestration of potential partners in the nucleus. These findings show that CdGAP acts as a negative regulator of mature cell-cell contacts in keratinocytes. Altogether, we concluded that CdGAP function is necessary for stabilization of mature junctions (rather than their formation) and its overexpression negatively affects mature contacts.

We next evaluated whether CdGAP down-regulation of E-cadherin was present in different tumour types. *ARHGAP31* (CdGAP) mRNA levels showed an overall negative correlation with total levels of E-cadherin protein in TCGA datasets, which was significant in stomach adenocarcinomas, ovarian cancer, colorectal adenocarcinomas, bladder and breast carcinomas (Fig. 1I; see methods). Higher levels of CdGAP mRNA expression correlated with decreased E-cadherin protein levels, supporting our data on the disassembly of mature junctions by CdGAP expression. These results indicate that CdGAP-dependent regulation of E-cadherin may be relevant for specific cancers.

### CdGAP-mediated junction disruption is GAP-dependent

**Figure 2:**
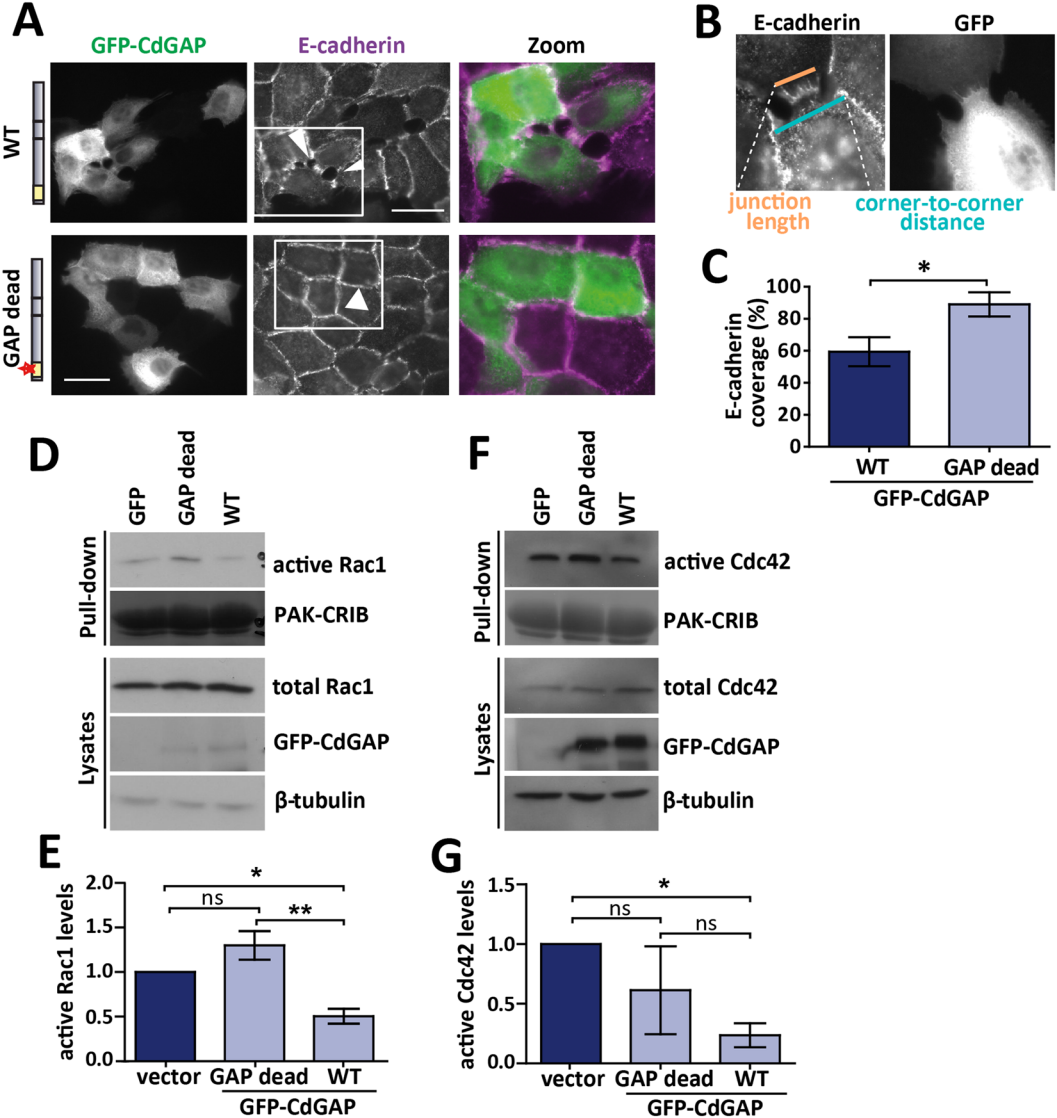
CdGAP-mediated junction disruption is GAP-dependent. Full length GFP-CdGAP wild-type or a GAP-dead CdGAP (catalytic dead) were expressed in keratinocytes with mature junctions for 8 hours (**A**–**C**) or 24 hours (**D**–**G**). (**A**) Cells were fixed and stained for E-cadherin. Images were collected and colour images from each channel overlaid to create merged images (Zoom). Arrowheads show junctions with interrupted E-cadherin staining. (**B**) Method used to quantify junction disruption. A parameter “E-cadherin coverage” is defined as the proportion of the length (pixels) of contact interface between neighbouring cells (corner-to-corner; blue line) that is covered by E-cadherin staining (orange line). (**C**) Graph shows the average E-cadherin coverage values following expression of CdGAP wild-type or GAP-dead. Values of around 100% coverage represent control junctions where E-cadherin staining extends from corner-to corner of a cell-cell contact. (**D**) Lysates of keratinocytes expressing different constructs were incubated with immobilised GST-PAK-CRIB to detect active, GTP-bound Rac1 (**D**) or Cdc42 (**F**, pull down). Total Rac1 or Cdc42 (2% total lysate volume), GFP-tagged CdGAP and β-tubulin were detected in the lysates. (**E**+**G**) Fold activation of Rac1 (**E**) or Cdc42 (**G**) was quantified by dividing the active GTPase level (pull down) by the total GTPase protein level (lysate) and normalising this to the value of the control (GFP-vector). Statistical significance was assessed using Student’s t-test (**C**) or one-way ANOVA with Bonferroni’s multiple comparison test (**E**,**G**). Standard error of the means is shown. Ns = not significant. *P ≤ 0.05, **P ≤ 0.01. Scale bar = 50 µm. N = 3.

We next asked whether the disruption of mature junctions observed upon CdGAP expression requires its GAP activity. As seen previously (Fig. 1H), expression of GFP-tagged wild-type (WT) CdGAP induced gaps in E-cadherin staining and junction disruption (Fig. 2A). In contrast, catalytically inactive CdGAP (GAP dead) had no effect (Fig. 2A). To quantify the extent of junction perturbation, we defined a parameter “E-cadherin coverage”, which represents the percentage of the opposing membrane interface at contacts that is covered by E-cadherin staining (corner-to-corner, Fig. 2B; see methods). In cells without perturbed junctions, E-cadherin staining extended from corner-to-corner of neighbouring cells (E-cadherin coverage, Fig. 2B,C). Expression of the GAP dead form had little effect on the extent of E-cadherin at junctions (89% E-cadherin coverage vs 59% in wild-type-expressing cells; Fig. 2C). Thus, junction disruption induced by CdGAP expression requires a functional GAP domain.

To determine which GTPases participated downstream of CdGAP in keratinocytes, global levels of active Rac1 and Cdc42 were assessed in keratinocytes transfected with CdGAP wild-type or GAP dead by effector pull-down assays (Fig. 2D–G). Expression of wild-type CdGAP significantly reduced the active levels of Rac1 and Cdc42, suggesting that junction disruption may be a consequence of inactivation of either Rac1 or Cdc42 or both. These results are consistent with our previous FLIM data that CdGAP expression in keratinocytes inactivates Rac1 both in the cytosol and at the membrane^21^. For the remainder of this study, we focused on Rac1 activity and function, as in contrast to other epithelial cells, Cdc42 regulation of keratinocyte junctions is not known.

### The scaffold protein Ajuba binds to CdGAP but is not necessary for CdGAP localisation at cell-cell contacts

**Figure 3:**
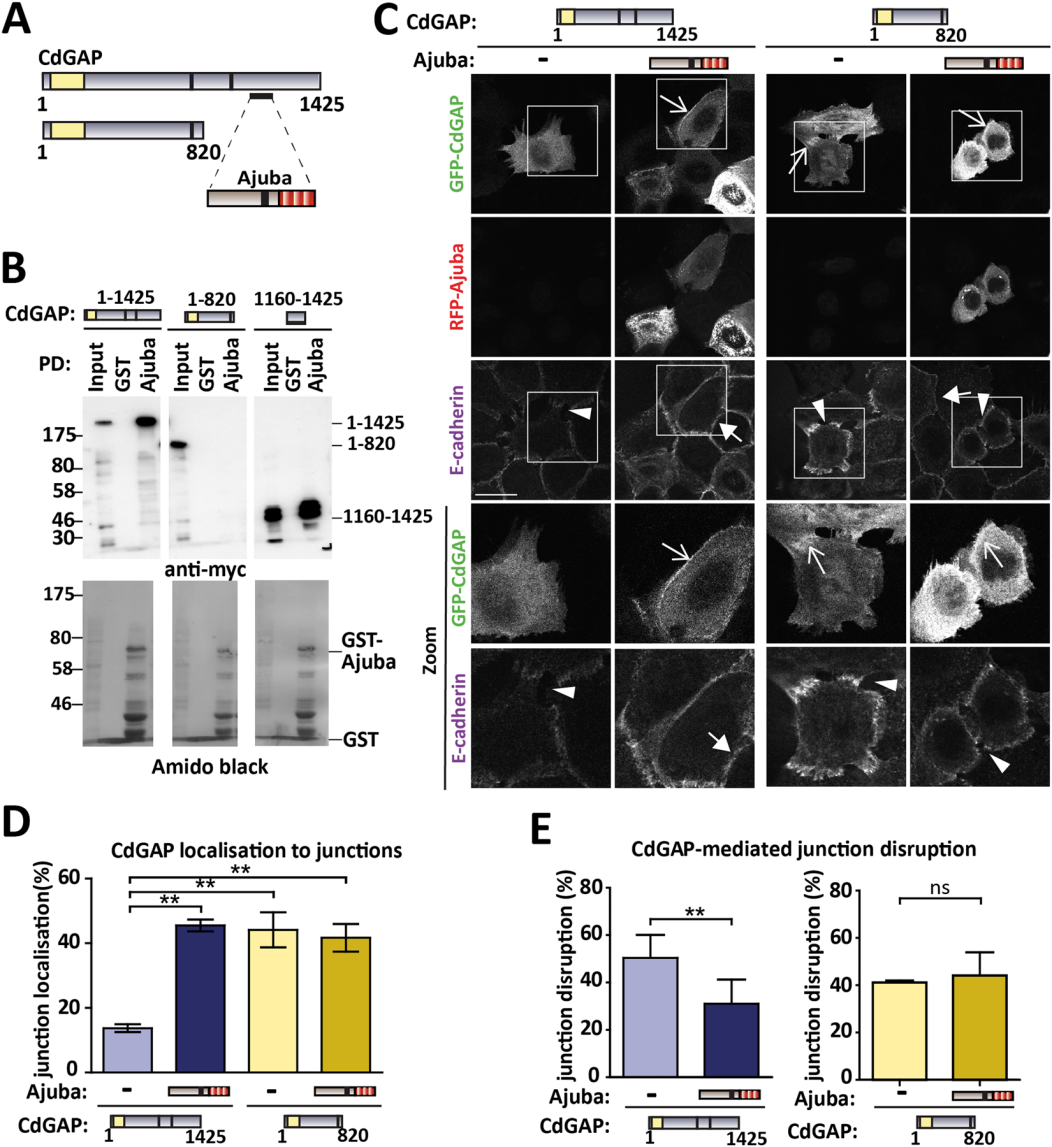
CdGAP localises to cell-cell contacts. (**A**) Summary diagram showing the potential Ajuba binding site on CdGAP based on yeast two-hybrid data (amino acids 1064 to 1183). CdGAP GAP domain is shown at the N-terminus, and proline-rich regions shown in blue. Ajuba LIM domains are shown in red and nuclear export sequence in black. Yeast two-hybrid assay detected a putative interaction between Ajuba and CdGAP. (**B**) COS-7 cells were transfected with myc-tagged CdGAP, CdGAP_1–820_ or CdGAP_1160–1425_ for 24 hours, lysates were used in pull down experiments with GST or GST-Ajuba full-length. (**C**–**E**) Keratinocytes with mature junctions were transfected with GFP-CdGAP or GFP-CdGAP_1–820_ in the presence or absence of RFP-Ajuba for 8 hours. (**C**) Cells were fixed and stained for E-cadherin. Images were collected on a confocal microscope and zooms are shown in bottom two rows. (**D**) The percentage of expressing cells with CdGAP enriched at junctions compared to the levels at the cytoplasm is shown. (**E**) Graphs quantify disrupted junctions in cells expressing the constructs outlined below the X axis. Disrupted junctions were qualitatively defined as those with two or more holes between neighbouring cells. Arrows and arrowheads indicate junctions with continuous or partially lost E-cadherin staining, respectively. Open arrows indicate regions enriched with CdGAP relative to the rest of the cell. Statistical significance was assessed using one-way ANOVA with Bonferroni’s multiple comparison (**D**) or Student’s t-test (**E**). Standard error of the means is shown. **P ≤ 0.01. Scale bar = 25 µm. N = 3.

To dissect further the CdGAP-dependent mechanism in epithelial cells, a yeast two-hybrid screen showed the potential binding of CdGAP C-terminus (1064–1183) to the LIM-domain protein Ajuba (Fig. 3A, A. Ferrand and D. Birnbaum, personal communication). This interaction was confirmed using pull-down assays: GST-Ajuba interacted with myc-tagged CdGAP, but not CdGAP_1–820_ (Fig. 3B). The C-terminal region of CdGAP (CdGAP_1160–1425_) was necessary and sufficient for Ajuba binding (Fig. 3B). As Ajuba localizes at cell-cell contacts where it modulates active Rac1 levels, the binding of CdGAP-Ajuba may be functionally relevant. We addressed (i) CdGAP intracellular localization, and (ii) whether Ajuba interaction was required for CdGAP localization or junction disruption (Fig. 3C–E).

Keratinocytes were transfected with CdGAP full length or CdGAP_1–820_ alone or in the presence of full-length Ajuba and images for E-cadherin staining and expression of the constructs were obtained (Fig. 3C). The percentage of junctions labelled with CdGAP was about 14% and, in the presence of Ajuba, CdGAP enrichment at junctions increased substantially to 46% (Fig. 3D). In contrast, CdGAP_1–820_ was enriched in 44% of junctions when expressed by itself, and this value was unaffected by Ajuba co-expression (42%). As both CdGAP constructs localized at junctions, we concluded that (i) CdGAP localises at cell-cell contacts independently of Ajuba and (ii) a pool of CdGAP full length requires Ajuba to be maintained at junctions, suggesting that Ajuba may play a role in the turnover of CdGAP at adhesion sites.

The Ajuba-dependent increase of CdGAP levels at cell-cell contacts poses a paradox: CdGAP expression negatively regulates cadherin adhesion, but Ajuba is necessary for junction maintenance. To investigate this further, the extent of junction disruption induced by CdGAP or CdGAP_1–820_ was quantified in the presence or absence of Ajuba (Fig. 3E; see materials and methods). Co-expression of Ajuba and CdGAP full length reduced the extent of disruption caused by CdGAP expression by itself (Fig. 3E). In contrast, the ability of CdGAP_1–820_ to perturb cell-cell adhesion was unaltered by the presence of Ajuba. Taken together, our data indicate that Ajuba may regulate both the junctional turnover and activation status of CdGAP full length, but not of CdGAP_1–820_.

### CdGAP and Ajuba interact directly via CdGAP C-terminus

**Figure 4:**
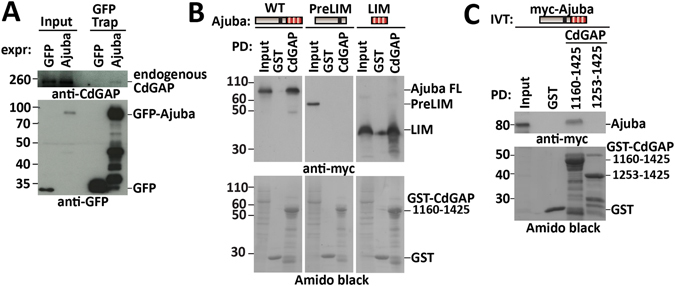
CdGAP and Ajuba interact directly. (**A**) Keratinocytes were transfected with GFP or GFP-Ajuba for 20 hours. Cells were lysed and incubated with GFP-trap to precipitate GFP-tagged Ajuba and associated endogenous CdGAP. Anti-GFP and anti-CdGAP antibodies were used to detect total and precipitated proteins. (**B**) Lysates prepared from COS-7 cells transfected with myc-tagged Ajuba full-length, preLIM or LIM domains were incubated with immobilised GST or GST-CdGAP_1160–1425_. (**C**) myc-Ajuba was produced via *in vitro* translation and incubated with immobilised GST, GST-CdGAP_1160–1425_ or GST-CdGAP_1253–1425_ for 2 hours. IVT = *in vitro* translated. Anti-myc detects associated proteins and expression levels (input). Amido black shows fusion proteins. Molecular weight markers are shown on the left of each panel. Input represents 5% of total lysate (**A**,**B**) or 20% of IVT protein (**C**). N = 3.

Expression of GFP-Ajuba was able to precipitate low levels of endogenous CdGAP (Fig. 4A), indicating the physiological relevance for a selected pool of endogenous CdGAP. The precise domain that mediates this interaction could be mapped further, since the minimal binding region of CdGAP (GST-CdGAP_1160–1425_) was able to pull down Ajuba full-length and the LIM domains (Fig. 4B). To determine a direct binding, we used two fragments (CdGAP_1160–1425_ and CdGAP_1253–1425_) to pull down *in vitro* translated full-length Ajuba (Fig. 4C). The longer fragment, CdGAP_1160–1425_, interacted with Ajuba, whilst CdGAP_1253–1425_ did not (Fig. 4C), suggesting that amino acids between 1160 and 1253 may be necessary for the interaction. We concluded that the C-terminal regions of both CdGAP and Ajuba bind directly with each other *in vitro* and in cells. Furthermore, the lack of interaction between CdGAP_1–820_ and Ajuba (Fig. 3B) is consistent with the fact that the ability to disrupt junctions of this CdGAP construct is unaffected by co-expression with Ajuba (Fig. 3D,E).

**Figure 5:**
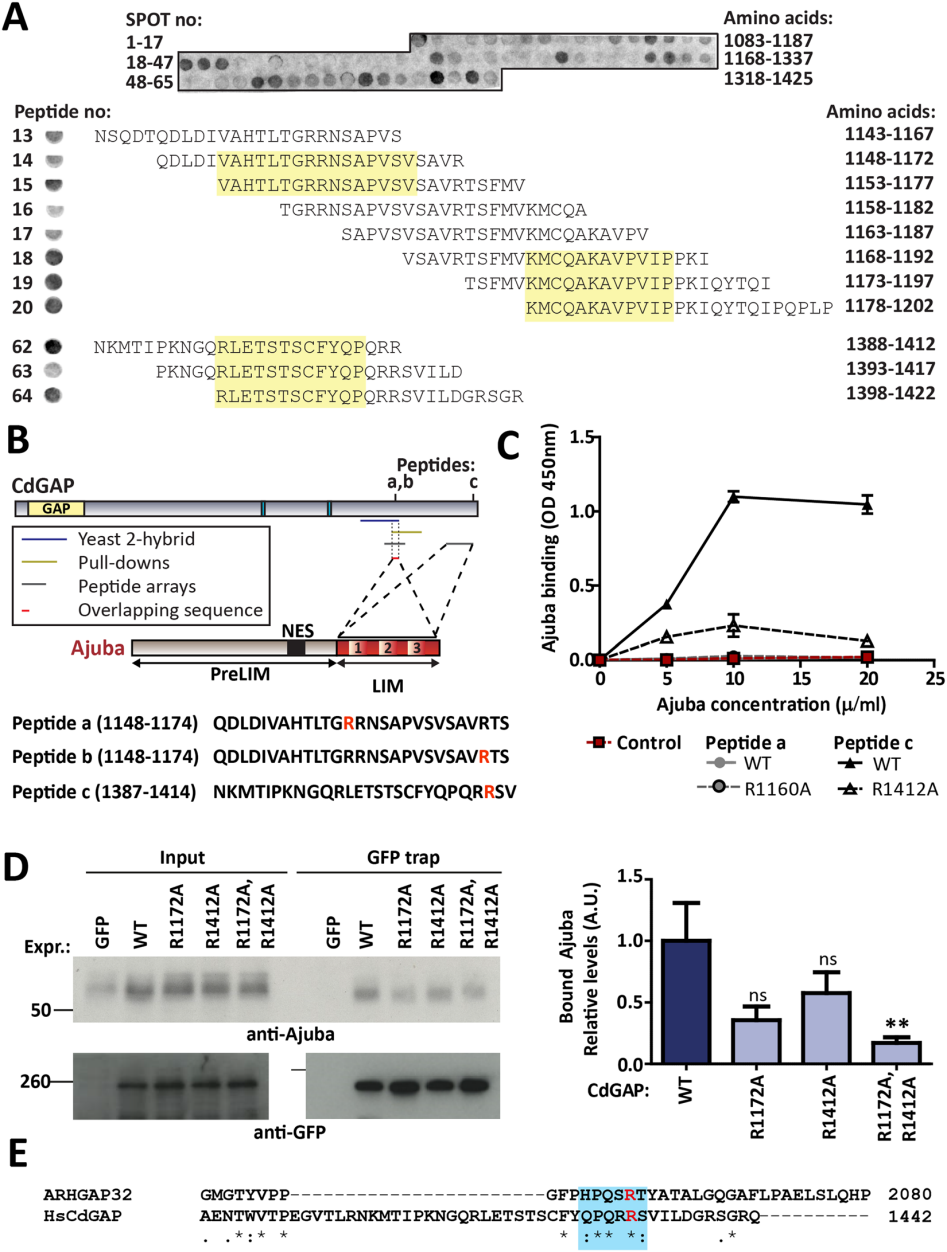
Conserved residues on CdGAP are responsible for the binding to Ajuba. (**A**) Peptides (25-mer) covering the C-terminus of CdGAP (amino acids 1083–1425) with 5 amino acid overlap were spot synthesised on a cellulose membrane and incubated with *in vitro* translated myc-Ajuba. Positive interactions were detected with an anti-myc antibody and visualised as a dark colour on the membrane. Peptide numbers along the CdGAP C-terminal sequence (Peptide no) and the amino acids contained in each peptide (Amino acids) are shown on the left and right hand side of the membrane, respectively (i.e. peptide number 1 contains amino acids 1083–1107). Sequences of highly reactive SPOTs are shown below the membrane and amino acid overlaps are shaded in yellow. (**B**) Overview diagrams of the interacting region between CdGAP and Ajuba to identify the minimal binding site on CdGAP: yeast two-hybrid (amino acids 1064 to 1183; navy blue line); pull-down assays (amino acids 1160 to 1253; yellow line) and peptide arrays (shorter peptides within the C-terminal region; grey lines). Selective reactive sequences from the peptide array (**A**) are listed below CdGAP diagram (peptides a–c). (**C**) Increasing concentrations of His-Ajuba were incubated with immobilized peptides on ELISA plates covering the wild-type CdGAP regions or with mutations at residues R1160A (peptide a) or R1412A (peptide c). (**D**) Keratinocytes were transfected with wild-type GFP-CdGAP or different mutants to test impaired binding to endogenous Ajuba. Cells were lysed and GFP-tagged CdGAP proteins precipitated (GFP-trap) and detected with antibodies as indicated in the blots. Quantification of the amount of co-precipitated Ajuba was normalized to the GFP-CdGAP found in the GFP-trap. (**E**) Alignment of the human CdGAP (ARHGAP31) and PX-RICS (ARHGAP32) sequences. Conserved arginine (1412 in CdGAP highlighted in red and surrounding conserved amino acids shaded in blue. (*), conserved residues; (:) conserved substitutions. Amino acid number is shown on the right. Statistical significance was assessed using non-parametric Kruskal-Wallis test and Tukey post-hoc test. **p < 0.009. Ns = not significant. N = 3 (**A**,**C**), N = 5 (**D**).

There is currently no 3D structure available for CdGAP. The CdGAP region that interacts with Ajuba is highly conserved across CdGAP orthologues from different species (Figure S2). However, it does not share similarity with other known proteins nor contain predicted domains. To ascertain regions of CdGAP important for mediating the CdGAP-Ajuba interaction, a library of overlapping 25-mer peptides shifted by five amino acids (amino acids 1083 to 1425 of CdGAP) was synthesized onto cellulose membranes and incubated with purified myc-Ajuba (Fig. 5A). At least three separate regions appear to be involved in Ajuba-CdGAP C-terminus interaction (Fig. 5A) and are summarized in Fig. 5B. Initially, we focused on two distinct regions around amino acids 1148–1174 (which overlaps with the region identified in Fig. 4C) and 1387–1414 of CdGAP because of the reproducibility of their interaction with Ajuba.

Amino acids from selected reactive spots identified in the peptide array were sequentially substituted to alanine residues (Figure S3). Three mutations were selected for further investigation: arginines at positions 1160 (R1160A, peptide a), 1172 (R1172A, peptide b) or 1412 (R1412A, peptide c) (Fig. 5B). To validate the importance of these residues in the CdGAP-Ajuba association, mutated and wild-type peptides were immobilized on ELISA plates and incubated with His-tagged Ajuba LIM domains (Fig. 5C). Peptide a did not interact with Ajuba, indicating that it may be a false positive. Unfortunately, peptide b was insoluble and could not be tested in this assay. In contrast, peptide c showed strong binding to Ajuba that was abolished upon mutation of arginine 1412 to alanine residue (R1412A), indicating the importance of this residue (Figure S3B).

To validate whether the identified amino acids were necessary for Ajuba interaction in the context of full length proteins in cells, we prepared GFP-tagged CdGAP full length harbouring individual mutations at arginines 1172 or 1412 (CdGAP^R1172A^ and CdGAP^R1412A^, respectively) as well as a double mutant (CdGAP^R1172A/R1412A^) (Fig. 5D). GFP-trap experiments were performed to assess binding of endogenous Ajuba. The interacting pool was corrected for the levels of CdGAP present in the GFP-trap and normalised to controls expressing wild-type CdGAP. The interaction of the CdGAP double mutant R1172A/R1412A with endogenous Ajuba was significantly lower than wild-type CdGAP. The single mutants followed the same trend, but their binding was more variable and values did not reach significance. Taken together, these data support the identification of two important arginine residues, R1172 and R1412, in the C-terminal region of CdGAP that mediate the interaction with Ajuba.

The identified regions (1148–1174) and (1387–1422) of CdGAP lie in two highly conserved CdGAP regions across different species (Figure S2), suggesting the evolutionary importance of Ajuba interaction for CdGAP function. Furthermore, the sequences of CdGAP and closely related protein ARHGAP32 (PX-RICS) were aligned to specifically identify similarities at the C-terminal region (Fig. 5E). Interestingly, arginine 1412 was found within a sequence of four conserved amino acids present in both family members, representing a conserved motif of (H/Q)PQXR(S/T).

### Ajuba regulates CdGAP activity

**Figure 6:**
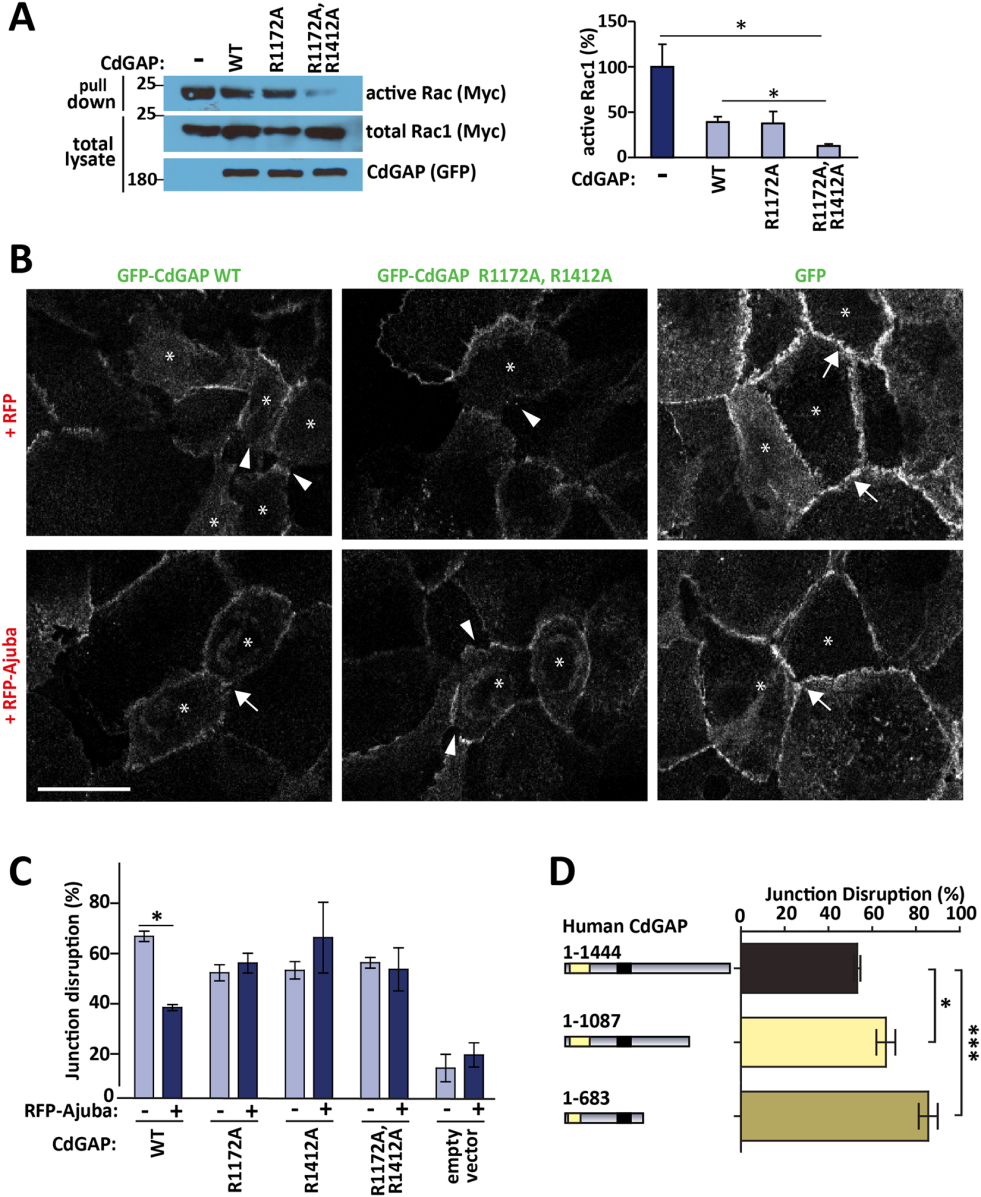
Ajuba regulates CdGAP activity. (**A**) Cells were co-transfected with myc-tagged wild-type Rac1 and GFP-tagged constructs of CdGAP wild-type (WT) or mutants with deficient Ajuba binding (R1172A and R1172A/R1412A). Lysates were prepared and incubated with GST-PAK-CRIB to detect active Rac1 (pull down). Total cell lysate shows levels of expressed proteins. (**B**) Keratinocytes with mature junctions were transfected with GFP-CdGAP wild-type or CdGAP mutant deficient in Ajuba binding, in the presence or absence of RFP-Ajuba for 24 hours. Cells were fixed and stained for E-cadherin. Images were collected on a confocal microscope (see also Figure S4). Arrows indicate junctions with continuous E-cadherin staining (arrows) or disrupted junctions with partial loss of E-cadherin (arrowheads). (**C**) The number of cells with highly disrupted junctions (defined as more than two holes between neighbouring cells) was counted for each condition. (**D**) Wild-type and truncation mutants of human CdGAP found in AOS patients were expressed in keratinocytes. The number of disrupted junctions was quantified as above. Scale bar = 25 µm. Statistical significance was assessed using two-way ANOVA (**C**) or one-way ANOVA (**A**,**D**). *P ≤ 0.05; ***P ≤ 0.001. N = 3 (**A**,**D**), N = 2 (**C**).

The direct association of Ajuba with CdGAP (Fig. 5) may facilitate the retention of the latter at junctions (Fig. 3). *In cellulo* data indicates that the presence of Ajuba may repress the ability of CdGAP to perturb cadherin adhesion (Fig. 3E). To explore whether Ajuba is able to inhibit the CdGAP activity, effector pull-down assays determined whether mutants with impaired ability to interact with Ajuba (CdGAP^R1172A^ or CdGAP^R1172A/R1412A^) have distinct GAP activity when compared with the wild-type protein. Active Rac1 levels following expression of wild-type CdGAP were significantly higher than upon expression of Ajuba-binding deficient double mutant (Fig. 6A). This result indicates that Ajuba binding at Arginines 1172 and 1412 on CdGAP C-terminus attenuates CdGAP activity.

The above CdGAP mutants were used to investigate whether Ajuba interaction with CdGAP is a key event to reverse the perturbation of mature cell-cell contacts (Fig. 3E). RFP-Ajuba was co-expressed with GFP-CdGAP wild-type or mutants with impaired ability to bind Ajuba, cells were stained for E-cadherin and the degree of junction disruption quantified (Fig. 6B, C, Figure S4A). Whilst co-expression of wild-type CdGAP with Ajuba partially rescued junction disruption, such rescue did not occur upon co-expression with any of the CdGAP mutants (R1172A, R1412A or double mutations). Taken together, our data strongly indicate that the direct interaction between Ajuba and CdGAP is essential to prevent keratinocyte junction destabilization.

Mutations leading to truncated forms of CdGAP (1–1087 and 1–683) have been identified as a causative factor in the developmental disorder AOS^9^. As these truncated forms of CdGAP lack the C-terminal region that interacts with Ajuba, we asked whether these mutants could influence junction stability. Upon expression of CdGAP truncation mutants 1–1087 or 1–683 in keratinocytes, severe disruption of E-cadherin contacts was observed and significantly higher when compared to expression of full-length CdGAP (Fig. 6D, Figure S4B). These results confirm that the higher catalytic activity of these mutants may severely affect tissue architecture in AOS patients. The significance of this finding is the potential contribution of disrupted cell-cell contacts to the pathology of the disease, in addition to the established role of migration.

## Discussion

**Figure 7:**
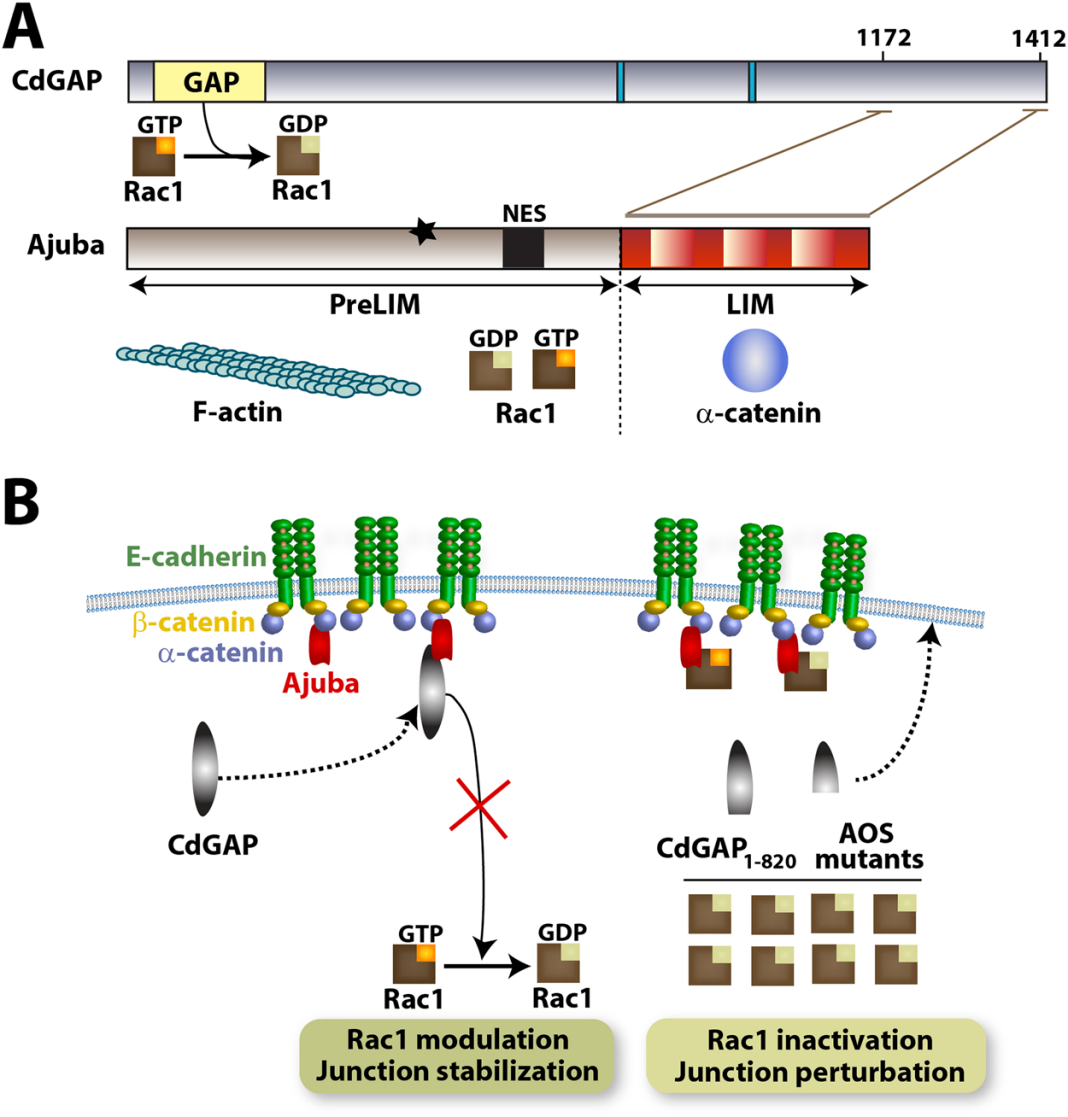
Summary of results. (**A**) Diagrams showing CdGAP and Ajuba domains, their respective partners and functional interaction with Rac1. CdGAP C-terminal residues R1172 and R1412 interact directly with Ajuba LIM domains. Ajuba LIM domains also associate with α-catenin, while PreLIM domains bind to F-actin. The preLIM domain also interacts equally with active or inactive Rac1, unless it is phosphorylated by PAK1 (black star) which increases its affinity for activated Rac1. **(B**) CdGAP is recruited to junctions via an unknown mechanism. CdGAP is maintained at cell-cell contacts via binding to Ajuba, which keeps CdGAP in an inhibited status, thereby preventing Rac1 inactivation locally. CdGAP_1–820_ and the AOS mutants lack the C-terminal region that interacts with Ajuba, have higher catalytic activity and stronger ability to perturb junctions.

Understanding how GAP proteins themselves are modulated is crucial to unravel the GTPase-dependent pathways governing cellular processes. Here we identify CdGAP as a novel regulator of Rac1 during the stabilization of mature cell-cell contacts in epithelia. CdGAP interacts directly with the LIM domain protein Ajuba, which is necessary for CdGAP retention at junctions in an inactive status (Fig. 7A). A unique aspect of our finding is the positioning of Rac1 at junctions in close proximity to its inactivator CdGAP, which is facilitated by their interaction via distinct domains of Ajuba. The potential retention of both Rac1^23^ and a Rac GAP at cell-cell contacts by Ajuba is a refined way to tune GTPase activity in a restricted area, thereby allowing for a fast and dynamic regulation of GTPase functions locally.

CdGAP function is not necessary for distribution of E-cadherin at newly formed junctions, but contributes to the resistance of cell-cell contacts to mechanical stress. In contrast, expression of either CdGAP or C-terminal truncation mutants potently disrupts mature cell-cell contacts in a GAP-dependent manner. Our results highlight the different cellular events and signalling processes that participate in assembly versus maintenance or stabilization of junctions^5^.

Ajuba is not necessary for recruitment of CdGAP (this work) or Rac1^23^ to cell-cell contacts. Rather, in the presence of Ajuba, full length CdGAP and active Rac1 are retained at junctions. It seems likely that Ajuba maintains CdGAP predominantly in an inactivated state at junctions (Fig. 7B). In spite of higher levels of CdGAP in the context of the junction milieu, junction perturbation is significantly attenuated upon co-expression with Ajuba. Furthermore, Ajuba depletion decreases Rac1 activation at cell-cell contacts^23^ and CdGAP mutants unable to interact with Ajuba show significantly higher catalytic activity on Rac1 in cells (this work)^9^.

The importance of the CdGAP-Ajuba interplay builds from our data that Ajuba LIM domains interact with CdGAP, whereas Rac1 binding occurs via the PreLIM domain (Fig. 7A)^23^. Conceivably, the same scaffolding protein may harbour an active GTPase (Rac1) and its GAP at junctions, thereby facilitating speedy Rac1 inactivation when necessary (Fig. 7A). Ajuba may thus have an important role to enable a precise control of Rac1 signalling at junctions in two ways: a favoured interaction with activated Rac1^23^ and spatial modulation of CdGAP activity to control discrete, fast and localized inactivation of Rac1 if required.

Our findings uncover general principles of GTPase regulation by proteins resident at cadherin-dependent cell-cell contacts^5^. First, interactions of GAPs or GEFs with junctional F-actin or actin binding proteins can provide a feedback mechanism that allows precise control of active GTPase. Similar sequestration and down-regulation of activity at junctions have been shown for the regulators Rho GAP p190B^27^, Rich1^28, 29^ and Trio^30^ or the GEF GEF-H1^31^. Second, interactions with proteins resident at cell-cell contacts may position GTPase regulators in well-defined regions along cell-cell contacts with clearly distinct functional outcomes. For example, the interaction at junctions of the Rac GEF Tiam1 with β2-syntrophin or the polarity complex Par3 may promote or inhibit its activity at the basal or apical regions or epithelia, respectively^32^.

Ajuba may also be required to regulate CdGAP activity in other cellular events where signalling is spatially confined. Although it has not yet been possible to determine the localization of endogenous CdGAP, Ajuba and Rac1 are found at cell-cell contacts, focal adhesions, nucleus and cytoplasm. Expression of CdGAP down-regulates Rac1 activity both at junctions and in the cytoplasm^21^. Clearly, distinct partners and regulatory mechanisms may play a role at these different intracellular locations. Nevertheless, CdGAP and Ajuba have been independently reported to participate in focal adhesion turnover, migration or cell proliferation^20, 25, 33, 34^. Furthermore, CdGAP transcriptional regulation of E-cadherin in breast cancers^15^ mirrors the well-established nuclear functions of Ajuba as a transcriptional co-repressor^35–38^.

The potential CdGAP-Ajuba cooperation in junction stabilization and in distinct cellular processes strongly supports a significant role for this partnership in epithelial morphogenesis and diseases^12^. CdGAP C-terminal truncation mutants found in AOS patients are hyperactive^9^, which is confirmed by their considerably higher disruption of keratinocyte cell-cell contacts when compared to wild-type CdGAP (Fig. 7B). Thus, CdGAP regulation of junctions may contribute towards a key developmental defect of AOS patients, the correct development of ectoderm and limb buds^9, 18, 19^, consistent with the developmental role of CdGAP substrates Rac1 and Cdc42^39–41^. Finally, in different cancers (this work)^15^, overexpression of CdGAP disrupts cell-cell adhesion and negatively correlates with E-cadherin protein levels. CdGAP modulation by Ajuba at cell-cell contacts would be particularly relevant via two mechanisms: preventing destabilization of junctions and cell detachment (this work) and decreasing the levels of the transcriptional repressors Snail and Zeb2, thereby promoting E-cadherin expression^15^.

In conclusion, we unveil a unique participation of Ajuba in maintaining CdGAP in an inactivated status to modulate Rac1 activity and stabilize junctions. This functional interaction between CdGAP and Ajuba highlights novel mechanisms via which GAPs are regulated in space and time. CdGAP joins the selective group of GAPs^5, 42^ that underpin tissue cohesion during epithelial tissue homeostasis, developmental pathologies and tumour progression. It can interfere with E-cadherin contacts by two mechanisms that are GAP-dependent (junction disassembly, this work) and GAP-independent (reduced E-cadherin transcription)^15^. CdGAP may be a tumour suppressor or tumour promoter, depending on its levels and alterations on its functional partners found in different tumours. Exciting new avenues are now opened to define how wide spread Ajuba regulation of other GAPs may be. Furthermore, future work will highlight whether CdGAP inactivation by Ajuba operates in other pathologies and identify potential mechanisms to modulate CdGAP function therapeutically.

## Materials and Methods

### Cell treatment, RNAi and transfections

Human keratinocytes isolated from neonatal foreskin were bought commercially (Lonza) and grown as described^23^: in standard calcium medium (mature junctions) or in low calcium medium to confluence, and then stimulating calcium-dependent adhesion by addition of calcium ions. Keratinocytes were transfected with plasmid DNA using JetPrime transfection reagent (Polyplus transfection), Viromer (Lipocalyx) or Fugene (Promega). siRNA oligonucleotides (Thermo Scientific or Eurogentecs) were transfected using INTERFERin (Polyplus transfection) according to the manufacturer’s instructions (CdGAP, 5′-GCTGTGACCTGACGGAGTA-3′ (oligo 2) and 5′-GGATGTAACCCATTCAGTA-3′ (oligo 3), Ajuba: D-021473-01 and D-021473-04, scrambled or non-targeting control oligo: D-001206-13). COS-7 and HEK293 cells were grown in DMEM supplemented with 10% fetal bovine serum, glutamine and the antibiotics penicillin and streptomycin. COS-7 cells were transfected when 70% confluent with lipofectamine (Invitrogen) according to the manufacturers’ instructions. Aggregation assays were performed with keratinocytes grown in low calcium medium essentially as described previously^43^.

### Constructs and mutagenesis

GFP-, myc-, and GST-tagged mouse CdGAP constructs have been described previously^7, 17, 44^. Human CdGAP constructs used the wild-type CdGAP protein (amino acids 1–1444) and the truncated proteins found in AOS patients (CdGAP 1–683 and 1–1087) have been described previously^7, 9^. GFP-CdGAP point mutants designed to perturb the binding of CdGAP to Ajuba were synthesised commercially in the mouse sequence CdGAP (CdGAP R1172A, CdGAP R1412A, and CdGAP R1172A/R1412A; GenScript). GST-PAK-CRIB and pRK5myc-Rac1 have been described^45^. Ajuba constructs were kind gifts from G. Longmore (Washington University): full-length Ajuba in pCS2-myc, mRFP1 or pGEX-2T and the truncation mutant pGEX-2T-PreLIM^25, 37, 46^. Full-length Ajuba was subcloned into pCS2-eGFP vector and Ajuba LIM domains were sub-cloned into pMSCG10-His tag vector. All constructs were verified by sequencing.

### Antibodies and microscopy

The following primary mouse monoclonal antibodies were used against: E-cadherin (HECD-1 kind gift from M. Takeichi), Myc (9E10, Sigma), actin (C4; MP Biomedicals), Rac1 (23A8; Millipore), RhoA (26C4, Santa Cruz), Cdc42 (44/CDC42, BD Transduction laboratories), β-tubulin (TUB 2.1, Sigma), GST (GST-2, Sigma), and anti-His conjugated to HRP (ab1187, Abcam). Rabbit polyclonal antiserum used were anti-α-catenin (VB1), anti-β-catenin (VB2)^47^, affinity-purified anti-Ajuba (4897; Cell Signaling Technology and 9104), anti-CdGAP^15^, anti-CdGAP (HPA036380, Protein Atlas, Sigma), anti-GFP (ab290, Abcam) and anti-Myc (A14, Santa Cruz). Secondary antibodies used were: fluorophore-conjugated (Jackson ImmunoResearch Laboratories, Inc.), horseradish peroxidase–coupled (Dako) and alkaline-phosphatase-conjugated antibodies (Sigma).

For immunofluorescence, cells were fixed with 3% paraformaldehyde (PFA) in phosphate-buffered saline (PBS) for 10 minutes at room temperate followed by permeabilization with 0.1% Triton X-100 and 10% FCS in PBS as described previously^48^. Images were acquired with an Olympus Provis BX51 microscope (60X magnification, 1.40 NA) coupled to a SPOT RT monochrome camera using SimplePCI 6 software (Hamamatsu, Japan) or with a Leica SP5 inverted confocal microscope (63x magnification, 1.40 NA) at our imaging facility (FILM, Imperial College London) using Leica LAS AF Lite software. Images were processed using Adobe Photoshop CS6 and WCIF ImageJ software.

### Determination of GTPase activity

Keratinocytes grown in standard calcium medium were transfected with different CdGAP constructs or empty vector control for 24 hours and processed for Rac1 effector pull down as described^45^.

### Pull-down assays

For pull-down assays, transfected COS-7 cells were washed in PBS then lysed in 250 µl of lysis buffer (10 mM Tris HCl pH 7.5, 150 mM NaCl, 1 mM MgCl_2_, 1% NP40 containing DTT, 1 mM each protease inhibitors and 10 mM each phosphatase inhibitors on ice, followed by centrifugation for 2 minutes at 4 °C at 14268.8 × *g*. Supernatant was incubated with GST or GST-fusion proteins. For *in vitro* pull-down assays, GST-tagged proteins, or GST alone immobilised on beads (2–5 μg) were incubated with myc-tagged proteins produced by *in vitro* translation. Samples were rotated at 4 °C and following washing in lysis buffer, resuspended in sample buffer and resolved by SDS-PAGE.

For the GFP-trap assay, cells were lysed after 20 hours transfection in 200 μl Ajuba lysis buffer (10 mM Tris pH7.5, 150 mM NaCl, 2 mM MgCl_2_, 1% NP40 containing protease and phosphatase inhibitors). Lysates were spun down at 14,268.8 × *g* for 5 minutes. Supernatants were diluted 5 times and added to GFP-trap beads (Chromotek) overnight at 4 °C. Beads were washed 3 times in lysis buffer without NP40 and bound protein analysed by Western blot.

### Peptide and protein production

Overlapping CdGAP peptides (25-mers frame-shifted by five residues) were synthesised in-house as spots onto cellulose membrane using the MultiPep SPOT synthesiser (Intavis AG). The sequence corresponded to amino acids 1083 to 1425 (mouse CdGAP sequence) and specific peptide sequences with demonstrated binding to Ajuba were further synthesized, where each residue was sequentially mutated to an Alanine. Soluble CdGAP peptides were synthesised in-house covering amino acids 1148–1175 and 1488–1414 either wild-type or with mutations R1160A, R1172A or R1412A.

GST-tagged fusion proteins were purified from *E. coli* using standard techniques. GST-Ajuba full-length was purified as described^23^. Proteins were *in vitro* translated using the TNT® Coupled Reticulocyte Lysate system (Promega) or in two separate kits, using the RiboMax kit (Promega) followed by the Retic lysate IVT^TM^ kit (Ambion) according to the manufacturers’ instructions. Protein synthesis was checked by SDS-PAGE followed by Western blotting.

### SPOT peptide array experiments

Spot array membranes were processed essentially as described^49^. Briefly, following blocking, membranes were overlaid for 2 hours at 4 °C with *in vitro* translated Ajuba or Ajuba Pre-LIM in 10 mM Tris HCl pH 7.5, 150 mM NaCl, 1 mM MgCl_2_, 1% NP40, DTT and 1 mM protease inhibitors and 10 mM phosphatase inhibitors). Bound protein was detected using either alkaline phosphatase via the addition of alkaline phosphatase substrate ((bromochloroindolylphosphate (BCIP), thiazolyl blue tetrazolium bromide (MTT) MgCl_2_ in citrate-buffered saline (CBS)) for up to 60 min as described^49^. Alternatively, reactive spots were identified with Indocarbocyanine (Cy3)-conjugated antibodies followed by detection using the Ettan DIGE imager (GE Healthcare).

### ELISA

ELISA plates (Nunc) were coated overnight at 4 °C with peptides at 50 μM (CdGAP or controls), blocked in 1% BSA in PBS at room temperature and washed (PBS with 0.1% BSA, 0.01% Tween-20). Plates were incubated with increasing concentrations of His-tagged Ajuba LIM domains for one hour at room temperature, washed, and incubated with an anti-His antibody conjugated to HRP for one hour at room temperature. Binding was determined by incubation with the horseradish substrate 3,3′,5,5′-tetramethylbenzidine (TMB, Pierce). The reaction was stopped via the addition of 1M sulphuric acid and plates read using the TECAN plate reader at 425 nm.

### Tumour dataset analysis

RNA-sequencing and reverse phase protein array (RPPA) datasets for lung adenocarcinoma and lung squamous cell carcinoma from the cancer genome atlas project (TCGA) were obtained from the UCSC cancer browser (https://genome-cancer.ucsc.edu/) and cbio portal (http://www.cbioportal.org)^50, 51^. All analysis were performed with R 3.1.0. Spearman’s correlation coefficient was used to measure the correlation between E-cadherin protein and ARHGAP31 mRNA expression in a panel of 19 cancer types. For each Spearman’s test, p value was computed and adjusted for multiple comparisons using Benjamini & Hochberg procedure (FDR). Only correlations with adjusted p value < 0.05 were shown.

### Quantification and statistical analysis

The levels of GTP-bound Rac1, Ajuba and CdGAP were assessed by densitometry using Image J software. GTP-bound Rac1 or Cdc42 was normalized to total Rac1 or Cdc42, respectively, detected in total cell lysates and GFP-trap-bound Ajuba was normalised to the amount of GFP-CdGAP bound to the GFP-trap to determine relative Ajuba binding. Junctional proteins were quantified as above and normalised to loading controls. The level of each protein in CdGAP-depleted cells was then expressed relative to control siRNA-treated cells. Quantification of junction disruption in CdGAP-expressing cells was performed using Image J to measure in number of pixels the length of the contact area between two cells, and the corresponding length covered by E-cadherin staining. The ‘E-cadherin coverage’ measurement was determined by dividing the length covered by E-cadherin by the total length of the contact area. Qualitative analysis of junction disruption was based on ascribing a retractile phenotype if the cell possessed at least one highly retracted junction, characterised by having at least two holes between the cell and its neighbour. For the qualitative analysis of CdGAP enrichment at cell-cell contacts, a junction was classified as being enriched if CdGAP was present in a continuous line covering more than 30% of the junction (as determined by area where E-cadherin is present) and this line was of greater intensity than the adjacent area.

All experiments were performed with independent replicates as stated in figure legends. Significance was tested using Student’s T-test; one-way or two-way Analysis of Variance (ANOVA), Kruskal-Wallis test and Tukey post-hoc test or the Friedman test using Microsoft Excel, Matlab or GraphPad Prism software as specified in figure legends. Figures were made using Photoshop and Illustrator.

### Data Availability

Data and reagents shown herein are available upon request.

## Electronic supplementary material

supplementary information
