# Supplementary material for: The scaffold protein Ajuba suppresses CdGAP activity in epithelia to maintain stable cell-cell contacts
Source: Sci Rep. 2017 Aug 23;7:9249. doi: 10.1038/s41598-017-09024-4 (PMC5569031; doi:10.1038/s41598-017-09024-4)

The scaffold protein Ajuba suppresses CdGAP activity in epithelia to maintain stable cell-cell contacts

J. J. McCormack, S. Bruche, A. B. D. Ouadda, H. Ishii, H. Lu, A. Garcia-Cattaneo, C. Chávez-Olórtegui, N. Lamarche-Vane, and V. M. M. Braga

Supplementary information

Supplementary Information

Supplementary Figure 1:

A-**E**, Keratinocytes transfected with either non-targeting siRNA (Scr) or oligonucleotide specific for CdGAP were grown to form confluent monolayers, detached from tissue culture dishes, dissociated into a single cell suspension and cell suspensions placed into at least six hanging drops in a humid chamber per condition. Cells were allowed to aggregate for 2 hours in the presence of calcium ions, dissociated by pipetting and all the resulting disaggregates imaged. **A**) The mean number of total disaggregates formed post-disruption was calculated. The size of initial aggregates (B) and all disaggregates formed post-disruption (C) was measured. The size of disaggregates was normalised to initial aggregate size (representative experiment shown). **D**-**E**) The size distribution of all disaggregates were plotted on histograms for each experiment. Statistical significance was assessed using Student's t-Test (A-B) and Mann Whitney Test (C). *, $P \leq 0.05$, ***, $P \leq 0.001$.

Supplementary Figure 2: Alignment of CdGAP C-terminus from different species.

ClustalW alignment was generated of the CdGAP C-terminal sequence from various species. Regions shaded in blue were identified in the peptide array as potential Ajuba binding sites. Residues shown in red were found to be critical for mediating the CdGAP-Ajuba interaction. Asterisks (*) represent conserved residues, colons (:) represent conserved substitutions (amino acids with strongly similar properties) and full stops (.) represent semi-conserved substitutions (amino acids of weak similar property). Amino acid number is shown on the right of each sequence.

Supplementary Figure 3: Arginine residues 1172 and 1412 are critical for the CdGAP-Ajuba interaction.

Peptides (25-mer) of highly reactive SPOTs identified in the peptide array (Figure 5A) were synthesised with amino acids individually and sequentially mutated to alanine. Peptide 14, covering amino acids 1148-1172 of CdGAP (A) and peptide 62, covering amino acids 1388-1412 of CdGAP (B) were overlaid with *in vitro* translated myc-Ajuba for 2 hours and reactive spots detected with secondary antibody coupled to alkaline phosphatase. Positive interactions are indicated by darker colouration on the membrane. Background intensity was subtracted from the reactivity of individual SPOTs and reactivities were expressed relative to the wild-type CdGAP peptide (WT). Values were plotted with respective mutations highlighted on the X-axis. N=3

Supplementary Figure 4: Expression of human CdGAP wild-type and distinct mutants shown in Fig. 6.

Expression of different version of CdGAP proteins was performed in keratinocytes and cells were fixed and ells were fixed and stained for CdGAP, E-cadherin and DAPI for the nucleus. Images were acquired on a wide-field microscope and shown here. White squares delimit areas shown in zooms below. Arrowheads point to disrupted junctions.

A) Mouse CdGAP mutants unable to interact with Ajuba (R1172A or R1412A) were expressed in the presence of RFP or RFP-Ajuba. Asterisks shows co-expressing cells. Images of wild-type and double mutant are shown in Fig.6B; quantification of the data is presented in Fig.6C.

B) Wild-type and truncation mutant of Human CdGAP found in AOS patients were expressed in keratinocytes. Quantification of the data is shown in Fig.6D.

Scale bar = 50µm, N=3.

Supplementary Table 1

Cancer code	Cancer type
COAD	colon carcinoma
CESC	Cervical squamous cell carcinoma and endocervical adenocarcinoma
STAD	Stomach adenocarcinoma
ESCA	Esophageal carcinoma
UCEC	Uterine Corpus Endometrial Carcinoma
BRCA	Breast invasive carcinoma
HNSC	Head and Neck squamous cell carcinoma
PAAD	Pancreatic adenocarcinoma
PRAD	Prostate adenocarcinoma
BLCA	Bladder Urothelial Carcinoma
OV	High grade ovarian serous cystadenocarcinoma
THCA	Thyroid carcinoma
SKCM	Skin Cutaneous Melanoma
KIRP	Kidney renal papillary cell carcinoma
KIRC	Kidney renal clear cell carcinoma
GBM	Glioblastoma multiforme
LGG	Brain Lower Grade Glioma
LUSC	Lung Squamous Cell Carcinoma
LUAD	Lung Adenocarcinoma

Supplementary Figure 1

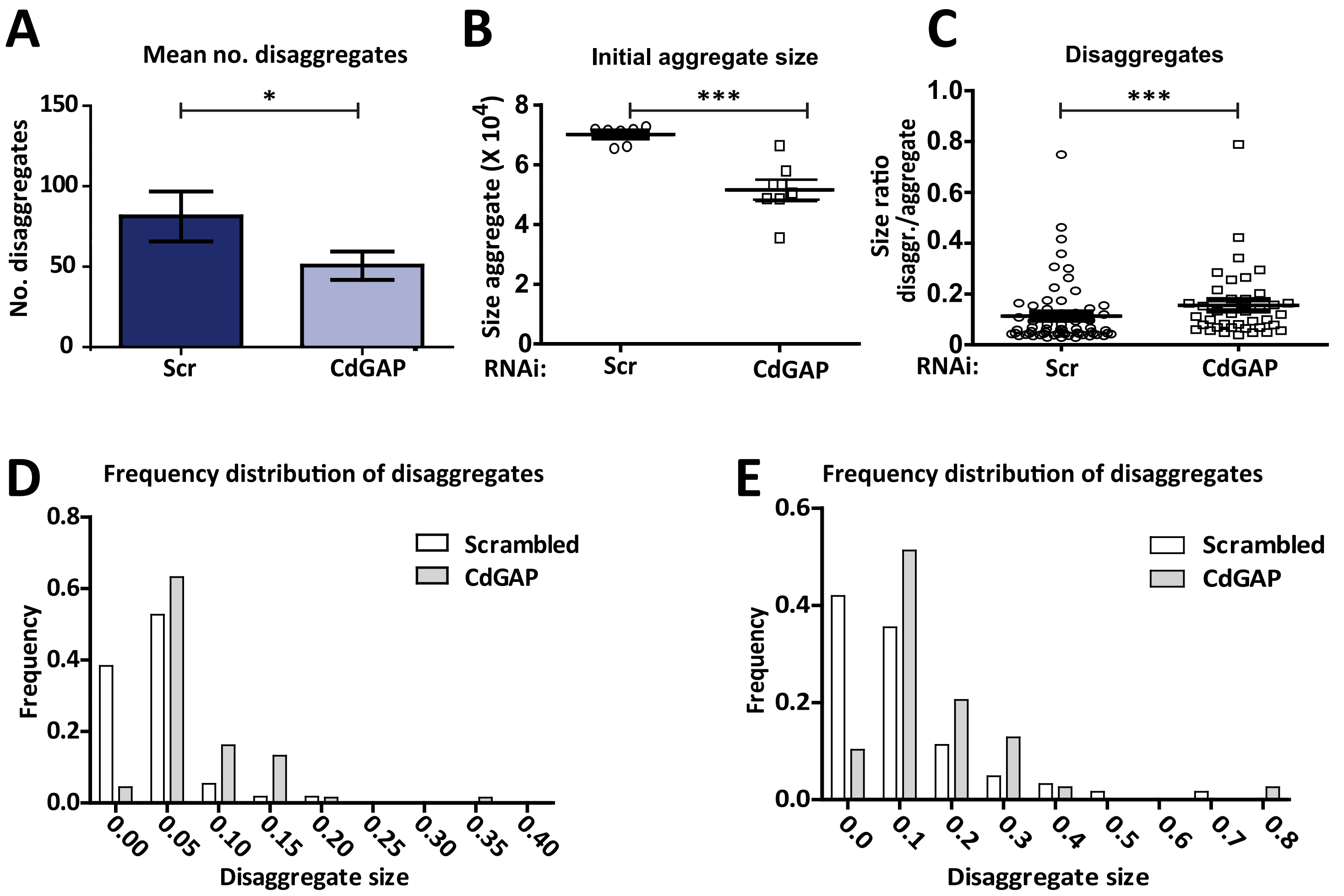

Supplementary Figure 2

```
Mus  >          -CKAAPWN--SQDTQDLDI----VAHTLTGRRNSAPVSVSAVRTSFMVKMCQAKAVPVIP 1189
Rattus>         -GKAAPWS--SQDTQDLGI----VAHALTGRRNSAPVSVSAVRTSFMVKMCQAKAVPVIP 1192
Homo>           -CKADPWRVYSQDPQDLDI----VAHALTGRRNSAPVSVSAVRTSFMVKMCQARAVPVIP 1204
Bos>            -GKVDPWRVYSQDSQDLDM----VAHALTGRRNSAPVSVSAVRTSFMVKMCQARAVPVIP 1211
Canis>          -CKVDPWSVCSQDPQDLDI----VVHALTGRRNSAPVSVSAVRTSFMVKMCQARAVPVIP 1212
Gallus>         GGKGVSWRSPFSAHVDLDLDYLAVAHATTGRRNSAPVSVSAVRTSFMIKMCQARAVPVIP 1191
                  *  .*    .   **.:    *.*: *******************:*****:******

Mus>            PKIQYTQIPQPLPSQSTGEGGAQPLERSQEEPGSTPEIPQKSTKDDSPSSLGSPEEEQPK 1249
Rattus>         PKIQYTQIPQPLPSQNTGEGGAQPPERNQEEPGSTPETPQKPTKDDSPSSLGSPEEEQPK 1252
Homo>           PKIQYTQIPQPLPSQSSGENGVQPLERSQEGPSSTSGTTQKPAKDDSPSSLESSKEEKPK 1264
Bos>            PKIQYTQIPQPLPSQSSEESGAQPLERNQEEAGSTGGTSQKLTKDDSLSSLESPREEKPK 1271
Canis>          PKIQYTQIPQPLPSQSSGDSGGQPLERSQEEPSSTGGTSQRSAREDSLS-LESPKEEKPK 1271
Gallus>         PKIQYTQIPQPLQAQNS----APQVEKKEAEAKQAGRQTQRVAWGHLEPPKSPLTEKKTK 1247
                ************ :*.:        *:.:  . .:   .*: :  .  .   .  *::.*

Mus>            QETGASASRRQASITSCMYEGSSCSPEPSASTLASTQDAVVQCRKRTSETEPSGDNLLSS 1309
Rattus>         QEQGASASRRKASTTSCVAEGSFCSAEPGPSTLVSSQDAMGQCRKRTSETEPSEDNLLSS 1312
Homo>           QDPGAIKSSPVDATAPCMCEGPTLSPEPGSSNLLSTQDAVVQCRKRMSETEPSGDNLLSS 1324
Bos>            QDTGAIESLPVDTTASHVCEGPTLPPEPGLANLLTTQDAVVQCRKRTSETEPSGDNLLSS 1331
Canis>          QDTGAIESLPMDTTTSHMCEGPTLPPEPVLANLLSTQDATVQCRKRTSETEPSGDNLLSS 1331
Gallus>         AEKENSDPAQKDSTCPWHS-SLVSPLESSQSSHNPSLDAPVLRRKRTSEGETGGDNPQSS 1306
                 :     .    :  .    .   . *.  :.  .: **    *** ** *.. **  **

Mus>            KLERASGGPKAFHRSRPGRPQSLILF----PIMDHLPSSPTVIDSKVLLSPIRSPTQTVS 1365
Rattus>         KLERASGGPKPTHRPRPGRPQSLILF----PIMDHLPSSPTVIDSKVLLSPIRSPSQTVS 1368
Homo>           KLERPSGGSKPFHRSRPGRPQSLILFSPPFPIMDHLPPSSTVTDSKVLLSPIRSPTQTVS 1384
Bos>            KIERSSGGSKPFHRSRPGRPQSLILFSPPFPIMDHPPPSSTGTDSKVLLSPIRSPTQTIS 1391
Canis>          KIERPSGGSKPFHRSRPGRPQSLILFSPPFPIMDHPPPSSTVTDPKVLLSPIRSPTQTVS 1391
Gallus>         KMERPSGFSKPSYRSRPGRPQSLILFSPPFPIMDHPSSS---ADSRVLLSPIRSPTQTSS 1363
                *:**.** .*. :*.***********    ***** ..*    *.:*********:** *

Mus>            PGLLCGELAENTWITPEGVTLRNKMTIPKNGQRLETSTSCFYQPQRRSVILDGRSGRQIE 1425
Rattus>         PGLLCGELAENTWVTPEGVTLRNKMTIPKNGQRLETSTSCFYQPQRRSVILDGRSGRQIE 1428
Homo>           PGLLCGELAENTWVTPEGVTLRNKMTIPKNGQRLETSTSCFYQPQRRSVILDGRSGRQIE 1444
Bos>            PGLLCGELAENTWVTPEGVTLRNKMTIPKNGQRLETSTSCFYQPQRRSVILDGRSGRQIE 1451
Canis>          PGLLCGDLAENTWVTPEGVTLRNKMTIPKNGQRLETSTSCFYQPQRRSVILDGRSGRQIE 1451
Gallus>         SSPICGDLSETSRTTPEGVMLRNKMTIPKNGQRLETSTSCFYQPQRRSVILDGRSGRQIE 1423
                .. :**:*:*.:  ***** ****************************************
```

Supplementary Figure 3

A

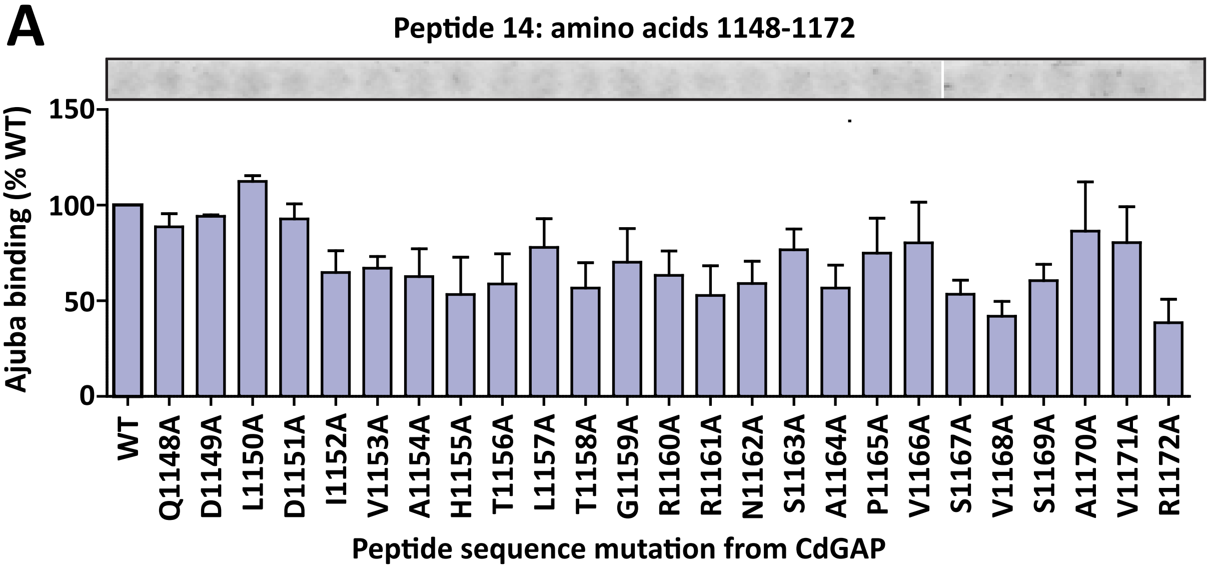

B

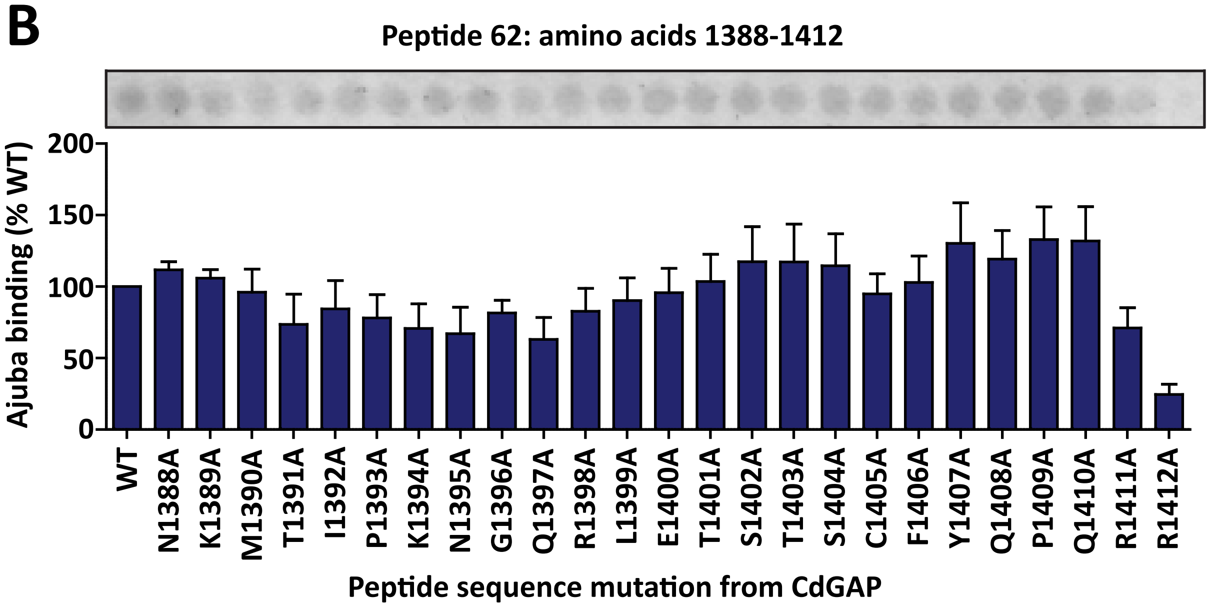

Supplementary Figure 4

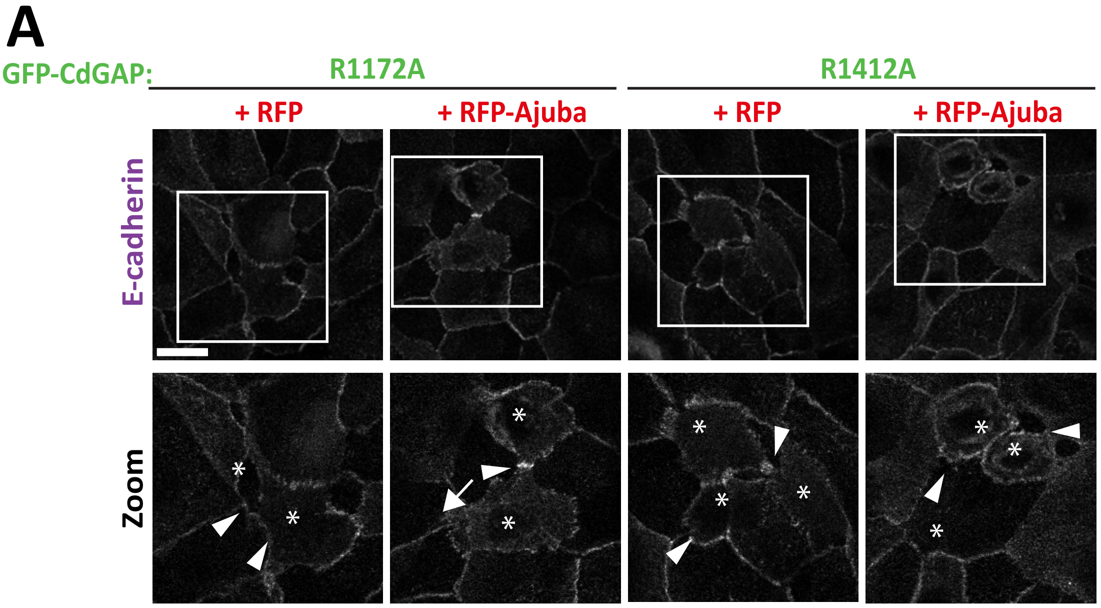

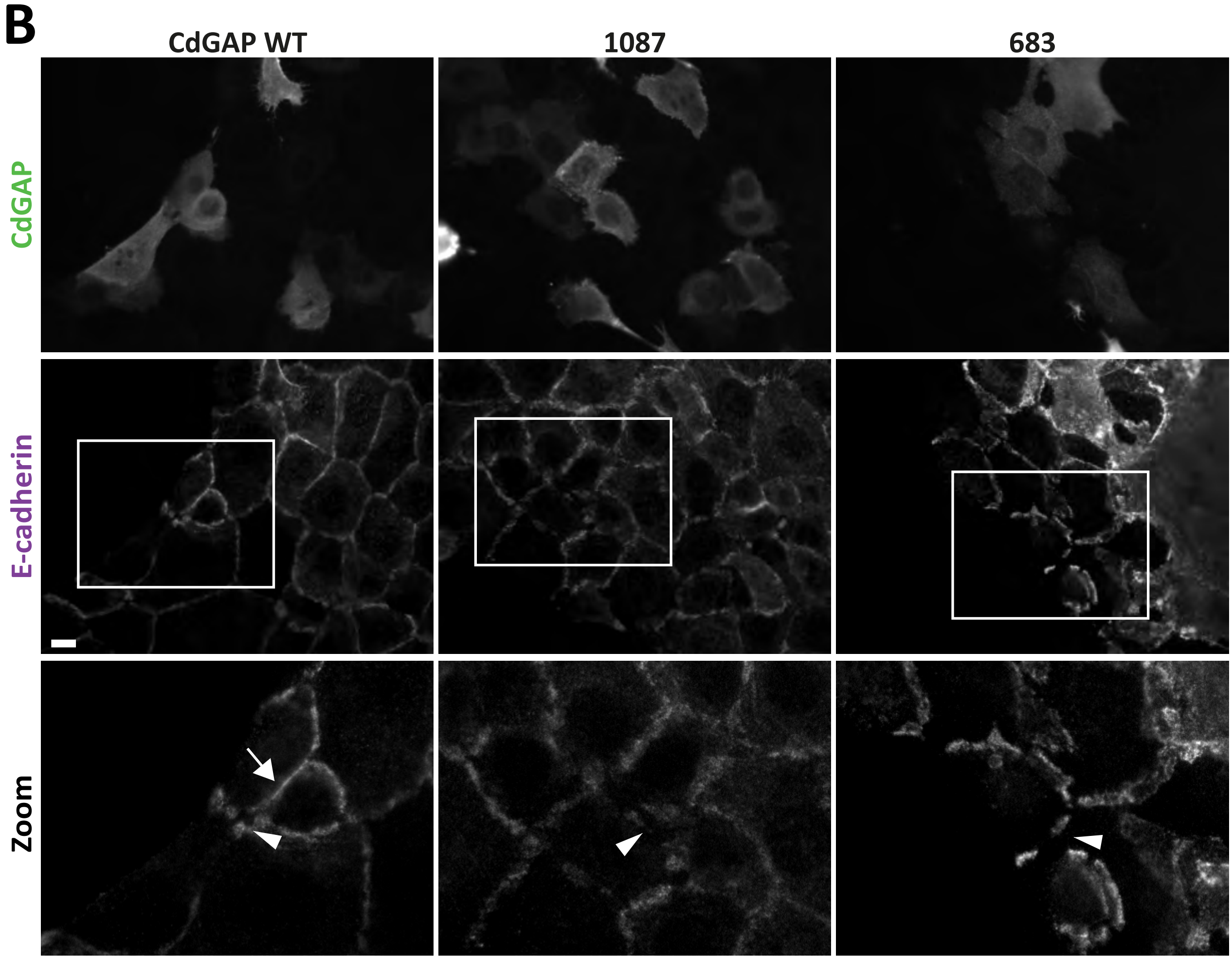

Supplement: Supplementary file 1 — supplementary information [file 41598_2017_9024_MOESM1_ESM.pdf]
